# Club Cell Secreted Protein CC16: Potential Applications in Prognosis and Therapy for Pulmonary Diseases

**DOI:** 10.3390/jcm9124039

**Published:** 2020-12-14

**Authors:** Sultan Almuntashiri, Yin Zhu, Yohan Han, Xiaoyun Wang, Payaningal R. Somanath, Duo Zhang

**Affiliations:** 1 Clinical and Experimental Therapeutics, College of Pharmacy, University of Georgia and Charlie Norwood VA Medical Center, Augusta, GA 30912, USA; salmuntashiri@augusta.edu (S.A.); yinzhu@augusta.edu (Y.Z.); yohan@augusta.edu (Y.H.); sshenoy@augusta.edu (P.R.S.); 2 Center for Vaccines and Immunology, University of Georgia, Athens, GA 30602, USA; xiaoyunwang@uga.edu; 3 Department of Medicine, Augusta University, Augusta, GA 30912, USA

**Keywords:** Clara cell, SCGB1A1, lung injury, COPD, bronchopulmonary dysplasia, sarcoidosis, idiopathic pulmonary fibrosis, respiratory infection, asthma

## Abstract

Club cell secretory protein (CC16) is encoded by the *SCGB1A1* gene. It is also known as CC10, secretoglobin, or uteroglobin. CC16 is a 16 kDa homodimeric protein secreted primarily by the non-ciliated bronchial epithelial cells, which can be detected in the airways, circulation, sputum, nasal fluid, and urine. The biological activities of CC16 and its pathways have not been completely understood, but many studies suggest that CC16 has anti-inflammatory and anti-oxidative effects. The human CC16 gene is located on chromosome 11, p12-q13, where several regulatory genes of allergy and inflammation exist. Studies reveal that factors such as gender, age, obesity, renal function, diurnal variation, and exercise regulate CC16 levels in circulation. Current findings indicate CC16 not only may reflect the pathogenesis of pulmonary diseases, but also could serve as a potential biomarker in several lung diseases and a promising treatment for chronic obstructive pulmonary disease (COPD). In this review, we summarize our current understanding of CC16 in pulmonary diseases.

## 1. Introduction

Millions of people suffer from respiratory diseases, a leading cause of death worldwide [1]. Even though significant progress has been made in the long-term management of chronic lung diseases, reliable tools for early diagnosis and effective therapeutic drugs are lacking [2]. Acute lung diseases such as acute lung injury (ALI) and acute respiratory distress syndrome (ARDS) are difficult to treat due to their sites of injury and inflammation are located deep in the lung [3]. It has been reported that bronchiolar destruction decreases lung function, leading to the development of chronic obstructive pulmonary disease (COPD), which especially targets the terminal bronchioles [4]. Currently, numerous studies are focused on lung epithelial cells to improve the outcome of acute and chronic lung diseases [5,6].

Club cells formally called Clara cells or bronchiolar exocrine cells, are non-ciliated bronchiolar secretory cells in the respiratory epithelium [7]. Approximately 8–14% and 17–27% of club cells are localized in the terminal bronchioles and respiratory bronchioles in humans, respectively [8]. In mice, the nonciliated cells comprise 50–70% of the total epithelial population throughout the entire airway epithelium, and >95% of them are club cells [9]. In the small airways, there are two subsets of secretory cells which recognized based on their vulnerability to naphthalene [10]. After naphthalene administration, the majority of those cells will be affected [10,11]. However, some secretory cells will survive and provide regenerative mechanisms in the damaged airway epithelium [11]. Subpopulations of club cells such as variant club cells are epithelial progenitors in airways after epithelium injury [11]. These cells are located near to neuroepithelial bodies or at the bronchoalveolar duct junctions, and it is characterized by the lack of cytochrome P450 expression, particularly CYP2F2 [10,11]. Club cells are heterogeneous, multifunctional cells, and have various physiological roles, such as secretion of defensive material [12]. In addition, they play an essential role in the repair process of the bronchial epithelium by acting as stem cells and providing biotransformation capacity, which usually enables the elimination of hazardous materials by detoxification [13]. After exposure to harmful factors, such as ovalbumin, they are quickly destroyed and are regenerated after about 30 days [12]. Furthermore, club cells play a key role in pulmonary homeostasis and immunity in both acute and chronic lung diseases. Club cells protect and regulate pulmonary function through the secretion of surfactants, glycosaminoglycans, enzymes, and other proteins [12].

CC16 is a protein primarily secreted by the club cells, which makes it a specific marker for these unique cells [14]. The dysregulation of CC16 was often observed in the pathogenesis of pulmonary diseases, such as ARDS, asthma, COPD, etc. Functionally, CC16 has been demonstrated to provide anti-inflammatory and anti-oxidative effects in various cells [15,16]. Considering its biological activities, many researchers are developing CC16 into a therapeutic agent against various respiratory illnesses. Given its essential roles, more research will be needed on CC16 to develop it into an early screening marker and a therapeutic agent for various lung diseases. In the current article, we review our current knowledge and the ongoing investigations on CC16 and its prospects in clinical medicine to prevalent or treat several acute and chronic lung diseases.

## 2. CC16 in ALI and ARDS

ALI and its advanced stage of ARDS are characterized by decreased lung compliance, severe hypoxemia, and bilateral lung infiltrate with high morbidity and mortality [17,18]. It may result from a variety of diseases including sepsis, pneumonia, trauma, etc. The severity and further features of ALI/ARDS may be detected by biomarkers in the circulation or bronchoalveolar lavage fluid (BALF). However, there is an urgent need for sensitive biomarkers that could identify patients who are at high risk of developing these disorders and differentiate these diseases from other causes of acute respiratory illnesses and non-pulmonary diseases [19].

Previous studies have reported that acute exposure to lung irritants causes elevated CC16 in the serum. For example, a temporary increase in serum CC16 was found in firefighters after 20 min of smoke exposure without any signs of lung impairment [20]. Another study showed that serum CC16 of cyclists who exposed to photochemical smog (O_3_ levels around 0.08) was slightly increased after two hours of riding. These findings of the increased concentration of serum CC16 in response to acute environmental changes suggest its potential utility as a reliable marker for the early detection of injury to acute airways and assessing the integrity of the lung epithelium [21,22].

In a clinical study, where 78 patients diagnosed with ARDS were examined to explore the potential correlation between serum CC16 levels and therapeutic outcomes, the median serum CC16 levels were higher in non-survivors and were associated with fewer days free of the ventilator (increased severity) [23], suggesting the potential use of serum CC16 in predicting the outcomes in ARDS patients who are at higher risk for mortality. Another study corroborated these findings with significantly higher CC16 serum levels observed in non-survivor ARDS patients compared to survivors [24]. In the latter study, there was a significant positive correlation between serum CC16 levels and Intensive Care Unit (ICU) stay, and a negative correlation with the PaO_2_/FiO_2_ ratio, an integral part of the assessment of patients with ARDS, thus demonstrating the potential utility of determining CC16 levels in the serum on ARDS severity and in distinguishing the various categories of ARDS. Similarly, Lin et al. reported plasma CC16 as a promising diagnostic biomarker to discriminate ALI from acute cardiogenic pulmonary edema [25]. Although the CC16 levels in the plasma or edema fluid were not a predictor of mortality in this study, these were associated with the duration of mechanical ventilation or ICU stay in ARDS patients compared to patients with acute cardiogenic pulmonary edema [25]. In addition to serum, increased CC16 levels in BALF has also been linked to ALI compared to non-smoking health subjects [26,27]. Intriguingly, the mean CC16 level in BALF from non-survivor ARDS patients was significantly lower than the survivors [26], suggesting the potential utility of measuring CC16 in BALF as a sensitive and specific biomarker to assess the role of the pulmonary epithelium in ALI [26].

Acute damage to club cells can be achieved experimentally using chemical pneumotoxicants such as methylcyclopentadienyl manganese tricarbonyl (MMT) or 4-Ipomeanol that cause a reduction in club cell numbers, CC16 mRNA, and protein levels in the lung [28]. In contrast, the serum concentration of CC16 is significantly increased due to leakage of the bronchoalveolar/blood barrier [28]. A transient elevation of CC16 in serum has been seen in rodents acutely exposed to O_3_ [21,22] despite the decreased production of CC16 from damaged club cells at high exposure levels. The elevation of CC16 serum was highly sensitive compared to albumin in BALF within 2 h of O_3_ exposure. These findings indicate that the assay of serum CC16 allows the accurate detection of even the minor defects in the epithelial barrier permeability. This higher sensitivity of CC16 most likely is because of its small size and high transepithelial concentration gradient compared to that of albumin. Consistently, Hantson et al. reported decreased serum CC16 levels after an initial transient increase compared with control rats exposed to chemicals [29]. A reduction in BALF CC16 and an increase in serum CC16 levels were reported in rats exposed to intratracheal lipopolysaccharide (LPS) [27]. This reduction was demonstrated, along with reduced CC16 messenger RNA expression in the lung, which was also seen in an acid aspiration rat model of ALI [30], suggesting that the decreased CC16 expressions in the damaged lung are common in various animal models of ALI.

In summary, acute exposure to lung irritants causes a transient increase in CC16 serum in both clinical and experimental studies as a consequence of increased airway permeability. In clinical studies, serum CC16 levels were reported to be higher when exposed to smoke inhalation or photochemical smog. The same findings were reported in rats acutely exposed to chemicals or O_3_. Furthermore, serum CC16 levels were higher in non-survivor ARDS patient samples compared to survivors and it was positively correlated with disease severity. In contrast, BALF CC16 levels were significantly lower in non-survivor ARDS patients than survivors. Consistently, BALF CC16 levels were lower in rats exposed to LPS and acid aspiration. In brief, there was consistency in the serum and BALF findings on CC16 levels between animal and human studies.

## 3. CC16 in Bronchopulmonary Dysplasia

Bronchopulmonary dysplasia (BPD) is a chronic inflammatory lung disease affecting premature infants characterized by impaired lung development, a need for supplemental oxygen, and long-term lung morbidity thereafter [31]. The assessment of disease severity is limited to parameters such as gestational age and requirement of supplemental oxygen [32]. Since these methods are time-consuming, there is a critical need for sensitive biomarkers that allow early diagnosis and management of BPD [33]. Analysis of mechanically ventilated neonates has indicated elevated serum CC16 levels in infants who developed BPD [34]. The increase in serum CC16 levels was particularly seen in neonates requiring ventilatory support as early as 2 h after birth indicating its prognostic utility. Interestingly, a similar study that examined cord blood CC16 in neonates with BPD indicated low cord blood CC16 levels as a predictor of BPD development in preterm infants [35]. Significantly lower levels of CC16 were also detected in BALF from ventilated neonates with BPD as compared to non-BPD neonates, indicating a positive correlation between BALF CC16 levels at birth and BPD diagnosis but a negative correlation with disease severity [36]. Furthermore, the authors suggested considering BALF CC16 levels as a biomarker for BPD at day 7 [36]. Taken together, previous studies have shown elevated serum CC16 concentration within 2 h after birth and a reduction in BALF CC16 concentration at day 7 among ventilated neonates who subsequently developed BPD.

## 4. CC16 in COPD

Chronic obstructive pulmonary disease (COPD) is the fourth leading cause of death in the US [37]. Long-term exposure to cigarette smoke (CS) coupled with genetic factors influences an individual susceptibility to develop the disease [38,39]. The severity and progression of COPD are determined by the forced expiratory volume/second (FEV1), and there are no definite biomarkers to identify people at risk for COPD before the onset of the disease [40]. Lower levels of CC16 in the circulation and airways have been associated with COPD prevalence and disease severity in clinical studies. In the Lung Health trial, a reduction in serum CC16 levels was associated with an accelerated decline in FEV1 over 9 years [41]. Furthermore, serum CC16 was the only biomarker found to be associated with a slower decline in FEV1 over 3 years in the Emergency treatment with Levetiracetam or Phenytoin in convulsive Status Epilepticus (ECLIPSE) trial [42]. Consistently, the same observations were seen in the Tucson Epidemiological Study of Airway Obstructive Disease (TESAOD), European Community Respiratory Health Survey (ECRHS), and Swiss Cohort Study on Air Pollution and Lung Diseases in Adults (SAPALDIA) trials over 14, 11, and 8 years, respectively [43]. Additionally, serum CC16 was measured in samples collected at ages 4–6 years in the Barn/children, Allergy, Milieu, Stockholm, Epidemiological survey (BAMSE), Manchester Asthma and Allergy Study (MAAS), and Children’s Respiratory Study (CRS) birth control trials, respectively, to examine the association of CC16 levels with subsequent lung function in early life [43]. They reported that a low level of CC16 at ages 4–6 years predicted subsequent FEV1 decline up to 16 years of age. The authors reported that decreased circulating CC16 levels are a risk factor for reduced growth and lung function in children, with a significant decline in lung function and airflow limitation in adults [43]. Moreover, airway CC16 expression was inversely correlated with the severity of airflow obstruction in COPD patients [44]. Consistently, patients with Global Initiative for Chronic Obstructive Lung Disease (GOLD) stages III–IV (severe and very severe, respectively) COPD had much lower airway CC16 expression than GOLD stages I–II COPD patients (mild and moderate, respectively) [45]. These observations are in agreement with another study reporting the complete disappearance of CC16 expression from the airways of severe COPD patients [46]. Strikingly, lower serum CC16 levels were detected in current smokers than former smokers with GOLD stage II–III, but not in stage IV, suggesting no correlation between serum CC16 levels and the severity of COPD [47]. In general, serum levels of CC16 were reported to be lower in current smokers than former smokers regardless of COPD diagnosis [47]. Furthermore, serum CC16 levels were significantly reduced in COPD patients than smoker and non-smoker controls [47]. Consistently, lower serum CC16 levels were detected in active smokers [41].

In animal studies, CS exposure gradually reduced airway CC16 expression in mice associated with pulmonary inflammation and COPD [40]. Mice deficient in CC16 had greater airspace enlargement, small airway remodeling, alveolar cell apoptosis, mucus metaplasia, and higher NF-kB activation than WT mice when both were exposed to CS for 6 months [45]. Consistently, in another study, CC16 deficient mice exhibited higher airspace enlargement and lung inflammation than WT when both were exposed to CS for 4 months [46]. In contrast, Park et al. reported that both WT and CC16 deficient mice developed comparable levels of airspace enlargement, emphysema, and small airway remodeling after 6 months of CS exposure [41]. It is important to note that the CS-exposure methods and duration were different in these studies, which may have contributed to the discrepancies in results. In non-human primates studies, CC16 airway expression was downregulated in two different monkey species, *Macaca mulatta* and *Macaca fascicularis*, after CS-exposure [46,48].

In summary, researchers observed lower CC16 expression during the pathogenesis of COPD in both preclinical and clinical studies. In human studies, serum CC16 levels were significantly reduced in COPD patients than smokers and non-smoker controls. Furthermore, CC16 was negatively correlated with accelerated decline in FEV1 in most longitudinal studies through-out childhood or elderly life, and it was significantly linked to disease severity. In animal studies, CC16 deficient mice showed higher airspace enlargement compared to WT from two different studies, and one study reported no differences between WT and deficient mice. In non-human primates studies, CC16 was down-regulated in two different monkey species after CS-exposure.

## 5. CC16 in Idiopathic Pulmonary Fibrosis

Idiopathic pulmonary fibrosis (IPF) is an epithelial-fibroblastic disease that primarily targets the alveolar epithelium in response to unknown factors. The main pathologic features of IPF are the loss of lung epithelial cells, aberrant tissue repair, activation of fibroblasts/myofibroblasts, excessive accumulation of extracellular matrix, and irreversible destruction of the lung parenchyma [49]. The diagnosis is clinically achieved with imaging tests and a biopsy, and the median survival rate after diagnosis is 3–5 years. Non-invasive biomarkers for the early diagnosis, differential diagnosis, prognosis, and prediction of therapeutic outcomes are extremely needed [50]. Serum and BALF levels of CC16 were significantly increased in IPF compared with non-IPF patients and healthy controls [50]. Tsoumakidou et al. observed increased CC16 levels in BALF and sputum supernatants from patients with IPF [51]. In addition, increased levels of serum CC16 were reported in patients with IPF alone and IPF combined with emphysema [52]. Consistently, elevated serum CC16 levels were associated with the involvement of pulmonary fibrosis in patients with systemic sclerosis [53]. Based on the published research articles, CC16 was significantly increased in biofluids, including BALF, serum, and sputum, in IPF compared with non-IPF patients and healthy controls.

## 6. CC16 in Sarcoidosis

Sarcoidosis is an inflammatory disorder of unknown etiology that is characterized by granuloma formation in affected organs, especially the lungs [54]. The disease course is highly variable and difficult to predict. Most cases report a marked remission rate, while persistent granuloma inflammation may lead to fibrosis or irreversible damage to other organs [55]. Diagnosis can be challenging because of the potential for organs other than the lungs to be influenced [56]. Thus, specific biomarkers with good sensitivity and specificity are needed to determine disease activity and to identify patients at risk of fibrosis. Previous studies have shown that serum CC16 levels were increased in individuals with sarcoidosis compared with healthy controls [57,58]. Consistently, Hermans et al. showed a considerable elevation of CC16 levels in serum, even in the absence of marked radiological abnormalities among sarcoidosis patients [59]. In the latter study, serum CC16 was significantly higher in patients diagnosed with sarcoidosis stages II–III than patients with stages 0–I. Interestingly, they found unchanged levels of CC16 in lung biopsies and correlated the elevated CC16 serum to the weak integrity of the blood–air barrier affected by the disease. Strikingly, the levels of CC16 in BALF were not markedly different between subjects, and not associated with the disease severity. Consistently, researchers found similar CC16 levels in BALF among subjects, and no differences between sarcoidosis stages [57]. In contrast, another study reported higher levels of CC16 in both serum and BALF from sarcoidosis patients than in healthy controls [58]. However, the reasons for this discrepancy are not clear. Taken together, serum CC16 rather than BALF CC16 was increased in individuals with sarcoidosis compared to healthy controls, and it was associated with severity. The high serum levels of CC16 may be correlated to epithelial-barrier leakiness. To be noted, these studies were performed in the early 2000s and no further investigation was published since then. Currently, CC16 is not considered as a potential biomarker for sarcoidosis or sarcoidosis severity due to the limited studies.

## 7. CC16 in Respiratory Infections

Among the many viruses and bacteria that cause respiratory infections, respiratory syncytial virus (RSV) is the most common respiratory pathogen in childhood, which also attacks the elderly population [60]. In general, RSV causes bronchiolitis that might rarely progress into severe conditions including pneumonia, respiratory failure, and death [61]. Johansson and colleagues found that infants with RSV infection had significantly higher serum CC16 levels compared with healthy controls [62]. However, serum CC16 levels were found to be similar in children infected with influenza or parainfluenza virus compared with healthy controls [62]. Recently, Egron et al. measured both serum and urinary CC16 from the infants aged under 1 year hospitalized for acute bronchiolitis and found a urinary increase in CC16 correlating to the severity of acute bronchiolitis, suggesting the potential utility of urinary CC16 as a biomarker in acute bronchiolitis [63]. In adults, it has been reported that athletes who suffer from recurrent respiratory infections had significantly lower serum CC16 than controls, which might lead to the increased susceptibility to respiratory infections in athletes [64]. Although a few studies have been done, the regulation and function of CC16 in response to respiratory infections and bacterial infections are largely unknown.

## 8. CC16 in Asthma and Allergy

Asthma is a complex respiratory disorder generally associated with allergic hypersensitivity characterized by chronic airway inflammation [65]. Using biomarkers for early prediction and prognosis has great clinical significance [66]. A mutation in the CC16 gene has been associated with an increased risk of asthma in childhood which followed by a significant decrease in serum concentration of CC16 [67,68]. Furthermore, circulating CC16 levels in asthmatic patients with a long duration of the disease (more than or equal to 10 years) was significantly reduced compared to those with a short duration (less than 10 years) [69]. In general, lower serum CC16 levels was associated with allergic sensitization and asthma. In contrast, Kurowski et al. reported no significant association between CC16 serum levels and asthma or allergic rhinitis among athletes [64]. The subjective diagnosis in their study and the exercise pattern could be reasons for the inconsistent results. 

Based on the different asthma conditions, serum CC16 levels were significantly decreased in patients with refractory and non-refractory asthma, and no differences were reported between the subjects [70]. Consistently, Shijubo et al. reported no significant difference in serum CC16 between atopic and non-atopic asthmatics [69]. These findings are in agreement with another study reporting no differences in CC16 serum levels between atopic and non-atopic patients [64]. Interestingly, fewer CC16 epithelial cells have been seen in the airways of asthmatic patients compared to the control group [58]. Additionally, the mean BALF CC16 concentration in asthmatic patients was significantly lower compared with the healthy controls [71]. Similar findings were also reported in another study indicating lower BALF CC16 levels in patients with refractory asthma and non-refractory asthma with no differences between asthmatic subjects [70]. In contrast, Emmanouil et al. found that patients with refractory asthma had higher CC16 levels in BALF than those with mild to moderate asthma [72]. 

Interestingly, there are also reports on CC16 measurements from sputum, nasal lavage fluid, and urine of asthma patients. Emmanouil et al. found higher CC16 levels in the sputum of asthmatics compared to controls [72]. In contrast, De Burbure et al. reported no difference in CC16 levels from the sputum of atopic asthmatics and controls [15]. A study examining CC16 levels in urine found a negative correlation with asthma in children. Urine CC16 levels were lower in asthmatic children compared to healthy controls associated with reduced forced vital capacity [73]. Two studies focused on allergic rhinitis found reduced CC16 levels in nasal lavage fluids compared to controls during the pollen season [74,75]. Together, the literature indicates a significant reduction in serum CC16 levels in asthmatic patients with a longer duration of the disease compared to those with a shorter duration. The majority of studies that measure CC16 levels in BALF of asthmatics reported considerably lower CC16 levels than the controls. Moreover, CC16 levels in serum and BALF showed no differences or association with the severity of asthma.

## 9. Summary, Conclusions, and Future Perspectives

Based on our current knowledge, although the club cells are very sensitive to pathophysiological alterations, the potential mechanisms are not fully explored. It is well-known that club cells are sensitive to environmental contaminants, such as naphthalene, 4-ipomeanol, and 3-methylindole, because they contain high levels of cytochrome P450 monooxygenases and flavin-containing monooxygenases [12,76,77]. Another possibility for the club cells to be sensitive to environmental changes could be that unlike the ciliated epithelial and goblet cells, club cells are non-ciliated and non-mucus secretary cells that allow pathogens and other harmful molecules to directly influence these naked cells. In general, the most fatal and rapidly progressive idiopathic adult lung diseases are associated with high levels of CC16 in serum and BALF (Table 1). Although the exact reasons are not clear, increased CC16 levels in the body fluids of these patients could be due to the rapid stimulation of the club cells that promote CC16 secretion, particularly in the early stages of lung impairment. Our views are supported by the literature presented in this article on the ARDS and pulmonary fibrosis. Since CC16 level is very sensitive to lung injury, we believe that the level of serum CC16 will be higher in patients with direct lung injury as compared to ARDS caused by indirect ways.

The club cells are involved in wound repair and become activated after alveolar injury [80]. However, the exact role of CC16 in alveolar wound repair is not extensively investigated. In injury resolution, studies have reported that club cells migrate and replace damaged alveoli in the lung [81,82]. Thus, the increased blood capillary permeability of the alveoli may facilitate the diffusion of migratory club cells secreted CC16 into circulation [83]. Additionally, we believe that since IPF affects mostly the alveolar epithelium, we can expect a higher level of CC16 in serum. In sarcoidosis, a granulomatous disease that affects the lung in more than 90% of cases [84], it mainly starts in response to inhaled antigens that stimulate the inflammatory cells including dendritic cells, alveolar macrophages, alveolar epithelial cells, and the lymphocytes which lead to the formation of sarcoid granulomas and fibrosis. Since 20–25% of sarcoidosis cases can develop pulmonary fibrosis at a later stage [85], we may expect to see higher CC16 levels from both diseases.

In contrast to the high circulatory CC16 in ALI/ARDS, COPD, and asthma studies report low levels of CC16 in the circulation and airways (Table 1). We correlated these findings to the nature of the diseases since they both progress in a long period. COPD is primarily caused by smoking, and there is a possible mechanism that could explain how club cells are affected by CS. During smoking, our bodies produce large quantities of oxygen-free radicals, in turn, activating oxidative stress pathways, which might damage the club cells resulting in reduced CC16 over time [46]. In asthma, one study reported a decrease in circulating CC16 level while T-cells, eosinophils, and mast cells were increased in small airways [86]. Future studies may explain if these cells can modulate or mediate CC16 protein. Even though most asthma cases reported lower levels of CC16, one study found that there is no difference between asthmatic and control among athletes. That could be because the CC16 level had been affected by exercise intensity. Additionally, most allergic rhinitis cases reported no difference in CC16 level compared to controls, probably because not all cases of allergic rhinitis have lung abnormality (40% of cases have asthma) [87]. In BPD, supplemental oxygen therapy in the preterm infants may accelerate the club cell death in addition to the main cause of BPD. Club cells are influenced by hyperoxia [88], and that could be a reason why CC16 level decreased and increased rapidly in the airway and the serum, respectively. Future studies may clinically correlate the damage of club cells to oxygen therapy in all patients requiring invasive or non-invasive mechanical ventilation.

Literature indicates that changes in CC16 levels can be measured from serum, BALF, sputum, nasal fluid, and urine. We believe that the reliability of measuring CC16 in the sputum and nasal lavage fluid has not been accurate enough in comparison to the serum or BALF. That is because nasal and sputum were not considered for CC16 measurements in most studies and when considered, the results were not comparable. Interestingly, CC16 from urine could be an accurate assessment for lung abnormality in children, such as asthma [73] and acute bronchiolitis [63]. Thus, we strongly recommend measuring CC16 from serum in adults and urine in children.

Even though CC16 has been affected by most pulmonary diseases and it could be used as a biomarker, several concerns remain. Firstly, the majority of studies did not correlate CC16 with disease severity. Although CC16 can be used to predict a lung abnormality, it cannot be used alone as a diagnostic tool since it is not a specific marker for one particular lung disease. Furthermore, the reference value for CC16 is still missing. Even though most studies reported a higher or lower level in a specific disease, the exact protein levels from each study were different as shown in Table 2. Hence, to accurately measure the CC16 concentration from healthy and sick people, we have to consider all factors that might affect CC16 levels such as age, gender, renal function, weight, and others is recommended. The reason why this protein has variable levels in circulation, as well as the airway, also needs to be investigated. Assays such as ELISA used for the CC16 measurements could have variability [45]. Hence, multiple measurements of CC16 in patient biopsies compared to healthy controls is recommended for accuracy.

In conclusion, circulating CC16 could help to diagnose and reflect the severity of a variety of lung diseases. However, the diagnosis cannot merely rely on the changes in CC16 levels in tissues or body fluids. The standard diagnostic tools such as spirometry and other pulmonary function tests are still needed. Furthermore, CC16 protein is very sensitive and many parameters such as gender, ethnicity, body-mass index, exercise, etc. should be considered for accuracy. Reports from Lesur et al. [23], Lin et al. [24], and Kropski et al. [64] have demonstrated that the CC16 levels were affected by disease duration and that CC16 concentration could be a more useful biomarker in acute cases rather than chronic diseases. Future studies might shed light on the non-invasive measurement of CC16, particularly in patients with critical conditions such as ARDS. Finally, CC16 might be assessed to predict responses after treatment and could be correlated with morbidity or mortality. In addition, serving as a biomarker, CC16 has also demonstrated the ability to become a potential treatment method for COPD. One of the most critical findings among these reports is that the serum CC16 level is significantly related to COPD. Most of the mentioned reports have shown positive results on bronchial epithelial cells isolated from COPD patients after therapy with recombinant CC16 [40,45]. However, most of the reports are based on data from patients with mild or moderate symptoms. Thus, it is difficult to conclude that CC16 treatment might be able to cure severe COPD cases. Additionally, since the primary cause of COPD is smoking and smokers exhibit low CC16 levels, we speculate that CC16 treatment could confer protection to smokers from developing COPD. However, the precise protective mechanisms of CC16 in COPD remain unclear. Future studies are warranted to identify how endogenous CC16 elicits its effects and what pathways and receptors could be involved in protecting the lungs by CC16 stimulation, so the exogenous CC16 can be applied clinically. Altogether, this review has summarized the relationship of CC16 with various pulmonary diseases, discussed the potential utility of CC16 as a biomarker, and reasoned why CC16 can be used as a therapy for several lung diseases. A shred of evidence has indicated that the CC16 might help to provide a new diagnostic method and help to classify different pulmonary diseases based on their different responses. Furthermore, CC16 may facilitate a novel therapeutic development for respiratory disease patients as well. In general, future studies of CC16 are expected to provide more information with the development of CC16 as a potential biomarker and a therapeutic agent for various lung diseases.

## Figures and Tables

**Table 1:** The Changes of CC16 in pulmonary diseases.

Disease	Specimen	Change of CC16	Conclusion	Reference
ALI/ARDS	Human; Serum	↑	Temporary increased in firefighters after 20 min of smoke inhalation Slightly increased after two hours of riding in cyclists who exposed to photochemical smog No signs of lung impairment were reported	[20,21,22]
ARDS	Human; Serum	↑	Higher in non-survivors compared to survivors Positive correlation with severity and mortality	[23,24]
ALI/ARDS	Mice/Rats; Serum	↑	A transient elevation of CC16 when animals exposed to O_3_ or treated with 4-Ipomeanol/alpha-naphtylthiourea	[21,22,29]
ARDS	Human; BALF	↑	BALF CC16 levels were higher compared to controls	[26]
ALI/ARDS	Rats; Airways	↑	Lower CC16 concentration after treatment with LPS Lower CC16 messenger RNA expression in an acid aspiration rat model of ALI	[27,30]
BPD	Human; Serum	↑	Higher CC16 levels in mechanically ventilated neonates who developed BPD	[34]
BPD	Human; Cord blood	↓	Lower CC16 concentrations from cord blood predicted the development of BPD in preterm infants	[35]
BPD	Human; Airways	↓	BALF CC16 level was lower in ventilated neonates who thereafter developed BPD	[36]
COPD	Human; Serum	↓	Associated with FEV1 decline in most longitudinal studies throughout childhood (BAMSE, MAAS, and CRS birth control studies) or elderly life (LHS, ECLIPSE, TESAOD, ECRHS, and SAPALDIA) Reduced in COPD patients than smoker and non-smoker controls Lower in current than former smoker	[41,42,43,47]
COPD	Human; Airways	↓	Airway CC16 expression is inversely correlated with the severity of airflow obstruction in COPD patients	[44,45,46]
COPD	Mice; Airways	-	CC16 deficient mice had greater airspace enlargement than WT after CS exposure	[45,46]
COPD	Mice; Airways	-	Both WT and CC16 deficient mice developed similar increases in airspace enlargement after CS exposure	[41]
COPD	Non-human primates; Airways	↓	CC16 expression was downregulated in two different monkey species, Macaca mulatta and Macaca fascicularis, after CS-exposure	[46,48]
PF	Human; Serum/Airways	↑	BALF and serum levels of CC16 were significantly increased in IPF compared to non-IPF patients and healthy controls	[50,52]
PF	Mice	-	CC16 deficient mice developed more severe PF when exposed to Bleomycin	[78]
Sarcoidosis	Human; Serum	↑	Serum and BALF CC16 increased in individuals with sarcoidosis compared with healthy controls	[57,58,59]
Sarcoidosis	Human; BALF	↑	BALF CC16 increased in patients with sarcoidosis compared to controls	[58]
Sarcoidosis	Human; BALF	No difference	CC16 in BALF was not affected by sarcoidosis	[57,59]
Respiratory syncytial virus	Human; Serum	-	Positive association between the serum levels of CC16 and IgG against RSV in atopic athletes compared to non-atopic athletes and healthy controls	[64]
Respiratory syncytial virus	Mice	-	Increased persistence of viruses and extended viral-specific gene expression were seen in CC16 deficient mice after RSV infection	[79]
Asthma	Human; Serum	↓	Mutation in CC16 gene increased risk of asthma in childhood CC16 levels decreased in asthmatic patients with a long duration of the disease compared to those with a short duration Significantly decreased in patients with refractory and non-refractory asthma	[67,68,69,70]
Asthma	Human; Serum	No difference	No significant association between CC16 serum levels and asthma among athletes	[64]
Asthma	Human; Airways	↓	Fewer CC16 epithelial cells in the airways of asthmatic patients compared to controls Lower CC16 concentration in BALF of asthmatics compared with control healthy subjects	[58,70,71]
Asthma	Human; Sputum	↑	Higher CC16 levels in sputum from patients with mild to moderate and refractory asthma compared to controls	[15,72]
Asthma	Human; urine	↓	Lower urine CC16 levels in asthmatics children compared to healthy children	[73]
Allergic rhinitis	Human; Serum	No difference	No significant association between CC16 serum levels and allergic rhinitis among athletes	[64]
Allergic rhinitis	Human; Sputum	No difference	No difference in CC16 levels between atopic rhinitis and non-atopic controls	[15]
Allergic rhinitis	Human; Nasal lavage fluids	↓	Reduction in the levels of CC16 in nasal lavage fluids compared with controls during the pollen season	[74,75]

Abbreviations: CC16, Club cell secretory protein; ALI, Acute lung injury; ARDS, Acute respiratory distress syndrome; ↑, increase; ↓, decrease; BALF, Bronchoalveolar lavage fluid; BPD, Bronchopulmonary dysplasia; COPD, Chronic obstructive pulmonary disease; FEV1, Forced expiratory volume in one second; WT, Wild type; IPF, Idiopathic pulmonary fibrosis; PF, Pulmonary fibrosis; BAMSE, Barn/children, Allergy, Milieu, Stockholm, Epidemiological survey; MAAS, Manchester asthma and allergy study; CRS, Children’s Respiratory Study; LHS, Lung Health Study; RSV, Respiratory syncytial virus; ECLIPSE, Evaluation of COPD Longitudinally to Identify Predictive Surrogate Endpoints; TESAOD, Tucson epidemiological study of airway obstructive disease; ECRHS, European community respiratory health survey; SAPALDIA, Study on air pollution and lung disease in adults.

**Table 2:** Serum CC16 concentration in various conditions or diseases.

Condition/Disease	CC16 Serum Concentration (ng/mL)	*p*-Value	Reference
ARDS	ARDS: 54.44 ± 19.62 ng/mL Non-ARDS: 24.13 ± 12.32 ng/mL	0.001	[24]
	Severe ARDS: 64.73 ± 14.42 ng/mL Mild ARDS: 48.17 ± 19.81 ng/mL Moderate ARDS: 57.35 ± 19.33 ng/mL	<0.05	
	ARDS: 22 ng/mL, IQR 9–44 Cardiogenic pulmonary edema: 55 ng/mL, IQR, 18–123	0.053	[25]
	Non-survivors: 22 ng/mL, IQR 7–50 Survivors: 20 ng/mL, IQR 10–38	0.99	
	Non-survivors: 19.93 ng/mL, IQR 11.8–44.32 Survivors: 8.9 ng/mL, IQR 5.66–26.38	0.01	[21,22]
ALI	Firefighters: 54.4 ± 34.9 ng/mL Controls:19.5 ± 11.7	0.04	[20]
	Cyclists: men: 12.3 ± 0.9 ng/mL; women: 11.9 ± 1.3 ng/mL Controls: men: 11.2 ± 0.8 ng/mL; women: 11.1 ± 0.6 ng/mL	0.01	[21,22]
Smoking/COPD	Never: 8.81 ng/mL; Former: 8.16 ng/mL; Current: 6.21 ng/mL	<0.0001	[43]
	Nonsmoker: 14.6 ± 5.0 ng/mL, smoker 11.3 ± 5.3 ng/mL	0.0001	[59]
	Active smokers: 3.10 ± 2.23 ng/mL Sustained quitters: 4.35 ± 2.72 ng/mL Intermittent quitters: 3.90 ± 2.43 ng/mL	0.0001	[43]
	Current and former smokers with COPD: 4.9 ng/mL Current and former smokers without COPD: 5.6 ng/mL Non-smokers controls: 6.4 ng/mL	<0.001	[47]
IPF	Controls: 5.67 ± 0.42 ng/mL Emphysema: 5.66 ± 0.35 ng/mL Combined pulmonary fibrosis and emphysema: 9.38 ± 1.04 ng/mL IPF: 22.15 ± 4.64 ng/mL	<0.05	[52]
	Controls: 10.7 ± 7.6 ng/mL Non-IPF: 23.1 ± 13 ng/mL IPF: 31.2 ± 10.8 ng/mL	<0.0001	[50]
PF/systemic sclerosis	Systemic sclerosis with PF: 90.8 ± 110.7 ng/mL Systemic sclerosis without PF: 42.1 ± 80.7 ng/mL	<0.01	[50]
Sarcoidosis	Sarcoid patients: 25.9 ± 16.2 ng/mL Controls: 13.9 ± 5.2 ng/mL	<0.05	[50]
Respiratory Infections	RSV group: 19.9 (4.6–56.1) ng/mL Influenza/parainfluenza group: 12.7 (7.2–36.0) ng/mL Healthy controls: 10.5 (4.0–125) ng/mL	<0.01	[21,22]
	Atopic athletes: 6.88, IQR 5.37–9.15 ng/mL Non-atopic athletes: 6.6, IQR 5.03–9.06 ng/mL	>0.05	[21,22]
	Athletes reporting frequent URIs: 5.57 ng/mL Control athletes: 7.03 ng/mL	0.01	
Asthma	Asthmatics children: 7.96 ng/mL; 95% CI =6.79–9.31 Non-asthmatic children: 9.98 ng/mL; 95% CI = 8.83–11.26	0. 006	[21,22]
	Asthmatic nonsmokers: 7.02 ± 3.05 ng/mL Controls: 11.7 ± 3.90 ng/mL	<0.0001	[21,22]
	Atopic asthmatics: 7.50 ± 3.38 ng/mL Nonatopic asthmatics: 6.32 ± 2.39 ng/mL	>0.05	
	Asthmatics, long duration of disease: 6.37 ± 3.11 ng/mL Asthmatics, short duration of disease: 7.88 ± 2.78 ng/mL	0.0106	

Abbreviations: IQR, Interquartile range; URI, upper respiratory infections.

## Abbreviations

ALI	Acute lung injury
ARDS	Acute respiratory distress syndrome
BALF	Bronchoalveolar lavage fluid
BAMSE	Swedish abbreviation for Children, Allergy, Milieu, Stockholm, Epidemiology
BPD	Bronchopulmonary dysplasia
CC16	Club cell secretory protein
COPD	Chronic obstructive pulmonary disease
CS	Cigarette smoke
CRS	Children’s Respiratory Study
ECLIPSE	Evaluation of COPD Longitudinally to Identify Predictive Surrogate Endpoints
ECRHS	The European community respiratory health survey
FEV1	Forced expiratory volume in one second
ELISA	Enzyme-linked immunoassay
GOLD	Global Initiative for Chronic Obstructive Lung Disease
ICU	Intensive care unit
IL-1β	Interleukin-1-beta
IL6	Interleukin 6
IPF	Idiopathic pulmonary fibrosis
IQR	Interquartile range
LHS	Lung Health Study
LPS	Lipopolysaccharide
MAAS	The Manchester asthma and allergy study
MMT	Methylcyclopentadienyl manganese tricarbonyl
NF-kB	Nuclear factor-kappa B
NHPs	Non-human primates
NLR	NOD-like receptors
PaO_2_/FIO_2_	Pressure of arterial oxygen to fractional inspired oxygen
PF	Pulmonary fibrosis
rCC16	Recombinant club cell secretory protein
RSV	Respiratory syncytial virus
SAPALDIA	The Swiss study on air pollution and lung disease in adults
TCRS	The Tucson children’s respiratory study
TESAOD	Tucson epidemiological study of airway obstructive disease
TLR	Toll-like receptor
TNFα	Tumor necrosis factor-alpha
URI	Upper respiratory infections
WT	Wild type
